# An esophageal-pleural fistula following transesophageal echocardiography-guided left atrial appendage closure: a case report

**DOI:** 10.3389/fmed.2026.1833714

**Published:** 2026-05-13

**Authors:** Libin Qiu, Jinpeng Liu, Haijing Fan, Hongxia Hao

**Affiliations:** 1 Structural Cardiology Department, Weifang People’s Hospital (The First Affiliated Hospital of Shandong Second Medical University), Shandong, China; 2 Department of Cardiology, Weifang People’s Hospital (The First Affiliated Hospital of Shandong Second Medical University), Weifang, China; 3 Department of Arrhythmia, Weifang People’s Hospital (The First Affiliated Hospital of Shandong Second Medical University), Weifang, China

**Keywords:** atrial fibrillation, esophageal perforation, esophageal-pleural fistula, left atrial appendage closure, transesophageal echocardiography

## Abstract

Periprocedural complications of left atrial appendage occlusion (LAAC) are varied and potentially life-threatening. We report a rare case of an iatrogenic esophageal-pleural fistula following a TEE-guided LAAC procedure. A 77-year-old woman with atrial fibrillation and a contraindication to long-term anticoagulation underwent successful LAAC. Her post-operative course was complicated by a pneumothorax and persistent pleural effusion. The diagnosis of an esophageal-pleural fistula was established after analysis of the chest tube drainage revealed food particles. Given her age and active infection, a non-surgical, conservative management strategy was adopted, involving complete gut rest, broad-spectrum antibiotics, thoracic lavage, and enteral nutrition support. The fistula closed after 52 days of conservative management, confirmed by follow-up imaging. This case underscores that while TEE-related esophageal injury is exceedingly rare, it must be considered in cases of persistent post-procedural pleural effusion. Furthermore, it demonstrates that in selected high-risk patients, a conservative approach can be a viable management option. The decision to perform a pre-procedural upper GI evaluation remains complex, given the very low incidence of this complication.

## Introduction

Percutaneous left atrial appendage closure (LAAC) is increasingly performed as an alternative to anticoagulation for stroke prevention in patients with atrial fibrillation (AF) (1). Periprocedural complications, including pericardial effusion, stroke, air embolus, etc., are a major concern limiting the application of LAAC (2). Fistulas are rare but represent a complex and often fatal complication of LAAC (3). Here, we report a case of an esophageal pleural fistula following LAAC.

## Case report

**Figure 1:**
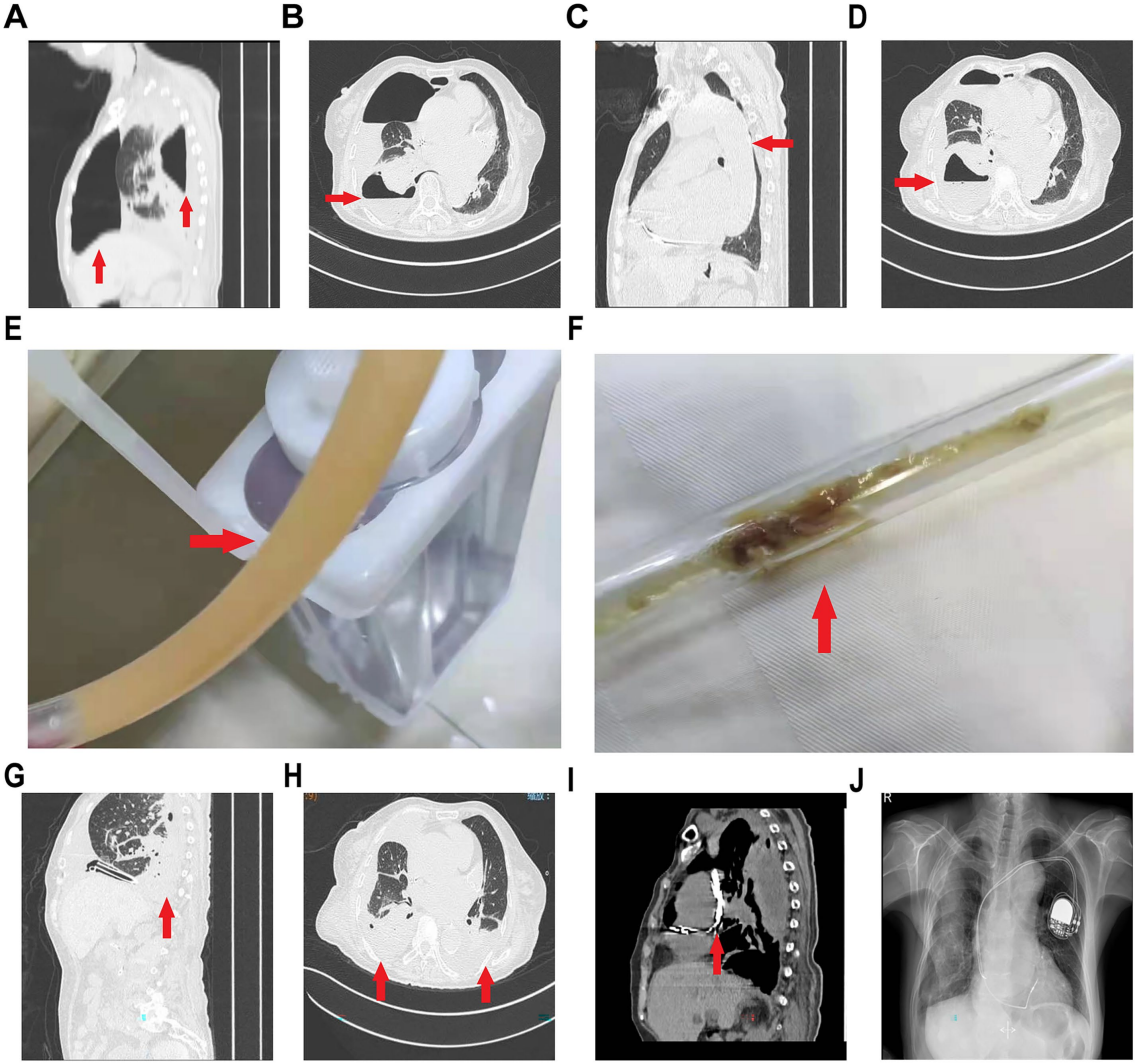
Serial thoracic imaging of the patient. **(A,B)** Day 1 post-procedure CT images (sagittal and axial views) demonstrate right-sided hydropneumothorax and pulmonary atelectasis. **(C,D)** Day 5 CT images (sagittal and axial views) show worsening right pleural effusion and consolidation. **(E,F)** Photographs of the chest tube drainage, which appeared turbid with foul odor and contained particulate debris. **(G,H)** Day 13 CT (sagittal and axial views) shows significant resolution of pleural effusion and pulmonary consolidation. **(I)** Day 52 contrast-enhanced upper gastrointestinal CT (axial view) confirms the closure of the esophageal-pleural fistula, with no evidence of contrast extravasation into the thoracic cavity. **(J)** Chest X-ray at 3 months shows complete pulmonary re-expansion. (Red arrow highlights the relevant pathological findings).

A 77-year-old woman with persistent atrial fibrillation and a high CHA₂DS₂-VASc score (female, >75 years, coronary atherosclerotic heart disease) and a history of gastrointestinal bleeding during oral anticoagulant therapy underwent percutaneous LAAC with a WATCHMAN device. The procedure was performed under deep sedation without endotracheal intubation and transesophageal echocardiography (TEE) guidance, though TEE probe insertion was notably challenging. Her post-procedural course was unremarkable, but on day 1, she developed chest tightness and dyspnea. Physical examination revealed diminished breath sounds in the right lung. Chest CT confirmed right-sided pneumothorax (Figures 1A,B), initially attributed to subclavian vein puncture during LAAC. Closed thoracic drainage was initiated, and she was managed with supplemental oxygen. However, the daily output remained persistently high (100–200 mL) with minimal symptomatic improvement. Given the clinical picture of persistent pleural effusion and pneumonia despite initial management with cefazolin, the treatment was escalated to empirical piperacillin-tazobactam combined with etimicin. By day 5, CT revealed worsening atelectasis and consolidation (Figures 1C,D). The drainage fluid became turbid and had a foul odor (Figures 1E,F). Analysis of the pleural fluid sediment surprisingly revealed food debris and vegetable matter, leading to a high suspicion of an esophageal-pleural fistula, potentially iatrogenic from the TEE probe. A multidisciplinary consultation was held immediately. The consensus was to manage the fistula conservatively without surgical repair, considering the patient’s age and active infection. A second chest tube was placed in the operating room to better drain the purulent cavity. Thoracic lavage was performed intermittently via the chest tubes with warmed sterile saline. An enteral feeding tube was placed in the interventional radiology department for post-pyloric feeding. The patient was transferred to the ICU for intensive management, which included the aforementioned antibiotic therapy, nutritional support (combined enteral and parenteral), and ongoing thoracic drainage with lavage. Notably, she did not require mechanical ventilation during her ICU stay. Absolute nil-by-mouth status, parenteral and late enteral nutrition, and gastrointestinal decompression were maintained. Gradual clinical improvement ensued. Follow-up CT on day 13 showed resolved pneumonia (Figures 1G,H). She was transferred back to the cardiology department for continued care. By day 52, contrast-enhanced CT confirmed fistula closure (Figure 1I). The patient resumed oral intake and achieved full pulmonary recovery within 3 months (Figure 1J).

## Discussion

This case highlights a rare but life-threatening complication of LAAC: an esophageal-pleural fistula. While LAAC is established for stroke prevention in atrial fibrillation patients contraindicated for anticoagulation, periprocedural risks such as pericardial effusion, device embolization, and vascular injury are well-documented (1). TEE guidance under deep sedation is the conventional modality for LAAC, used to evaluate device position and peri-device leaks. Esophageal injury of varying severity is a known risk, with rare cases presenting as perforation. Reported iatrogenic perforations during LAAC primarily involve cardiac structures, such as pericardial effusion/tamponade, or are device-associated, such as pulmonary artery injury (2, 3). Our patient embodied several high-risk features for TEE-related injury: advanced age (≥65 years), a recent history of gastrointestinal bleeding linked to anticoagulant use, and concurrent antiplatelet therapy. Contemporary data corroborate that patients undergoing TEE-guided transcatheter structural interventions, such as LAAC, represent a higher-risk cohort. Large-scale analyses report major TEE complication rates of 3.6% in these settings, significantly higher than in diagnostic examinations (4). Importantly, gastrointestinal hemorrhage constitutes the overwhelming majority (79–95.4%) of these complications, particularly in patients on antithrombotic therapy (4). In contrast, esophageal perforation or fistula formation remains a far rarer catastrophic event, with incidence estimates ranging from 0.01 to 0.38% in broader TEE studies, and a rate of 0.08% (approximately 1:1300) in a large prospective peri-operative audit (5, 6).

The non-specific presentation—initially masked by pneumothorax—underscores the need for heightened vigilance when post-LAAC respiratory distress persists despite standard interventions. Early recognition of atypical signs (e.g., feculent drainage and persistent pleural effusion) should prompt investigation for esophageal injury via contrast imaging. Management hinges on immediate gut rest, broad-spectrum antibiotics, and drainage of infected collections. While surgical repair is often advocated for overt esophageal perforations, conservative management sufficed in this case, aligning with evidence that select iatrogenic injuries can heal with non-operative support.

The extreme rarity of esophageal perforation raises the critical question of pre-procedural upper gastrointestinal tract evaluation. While our patient had identifiable risk factors, population-based studies indicate that the most severe TEE-related complications occur in patients without known pre-existing esophageal pathology. Furthermore, routine esophagogastroduodenoscopy prior to LAAC or similar procedures is not standard practice. Given the low incidence of perforation, the risks, resource utilization, and patient burden associated with subjecting all candidates—often elderly with multiple comorbidities—to an additional invasive screening procedure may not be justified (7). A preoperative chest CT, as performed in our case, is useful for assessing cardiac and aortic anatomy but has limited sensitivity for asymptomatic mucosal esophageal disease. Therefore, the decision for proactive endoscopic evaluation should likely remain individualized, based on specific symptoms (e.g., dysphagia and odynophagia) or a compelling history of esophageal disorder, rather than applied universally to all patients undergoing TEE-guided structural heart interventions.

To mitigate such risks, operators should prioritize gentle TEE manipulation, consider alternative imaging (e.g., intracardiac echocardiography (8)) in high-risk cases, and maintain a high index of suspicion for extrapulmonary complications when clinical trajectories deviate from expectations (9, 10). This case reinforces that even routine procedures demand meticulous technique and individualized risk assessment.

## Data Availability

The original contributions presented in the study are included in the article/supplementary material, further inquiries can be directed to the corresponding author.
